# Virulence Factors and Antimicrobial Resistance of Uropathogenic *Escherichia coli* EQ101 UPEC Isolated from UTI Patient in Quetta, Balochistan, Pakistan

**DOI:** 10.1155/2023/7278070

**Published:** 2023-09-11

**Authors:** Sareen Fatima, Ali Akbar, Muhammad Irfan, Muhammad Shafee, Amjad Ali, Zaara Ishaq, Syed Kashif Raza, Abdul Samad, Mohammad Y. Alshahrani, Syed Shah Hassan

**Affiliations:** ^1^Department of Microbiology, University of Balochistan, Quetta, Balochistan, Pakistan; ^2^Centre for Biotechnology and Microbiology, University of Swat, Charbagh, 19120 Khyber Pakhtunkhwa, Pakistan; ^3^Jamil-ur-Rahman Center for Genome Research, International Center for Chemical and Biological Sciences (ICCBS), University of Karachi, Karachi, Pakistan; ^4^Center for Advanced Studies in Vaccinology & Biotechnology (CASVAB), University of Balochistan, Quetta, Balochistan, Pakistan; ^5^Department of Industrial Biotechnology, Atta-ur-Rahman School of Applied Biosciences (ASAB), National University of Sciences and Technology (NUST), Islamabad 44000, Pakistan; ^6^Riphah International University Faisalabad, Pakistan; ^7^Department of Clinical Laboratory Sciences, College of Applied Medical Sciences, King Khalid University, P.O. Box 61413, Abha 9088, Saudi Arabia

## Abstract

Infectious diseases have been tremendously increasing as the organisms of even normal flora become opportunistic and cause an infection, and *Escherichia coli* (*E. coli* EQ101) is one of them. Urinary tract infections are caused by various microorganisms, but *Escherichia coli* is the primary cause of almost 70%–90% of all UTIs. It has multiple strains, possessing diverse virulence factors, contributing to its pathogenicity. Furthermore, these virulent strains also can cause overlapping pathogenesis by sharing resistance and virulence factors among each other. The current study is aimed at analyzing the genetic variants associated with multi-drug-resistant (MDR) *E. coli* using the whole genome sequencing platform. The study includes 100 uropathogenic *Escherichia coli* (UPEC) microorganisms obtained from urine samples out of which 44% were multi-drug-resistant (MDR) *E. coli*. Bacteria have been isolated and antimicrobial susceptibility test (AST) was determined by disk diffusion method on the Mueller-Hinton agar plate as recommended by the Clinical and Laboratory Standards Institute (CLSI) 2020, and one isolate has been selected which shows resistance to most of the antibiotics, and that isolate has been analyzed by whole genome sequencing (WGS), accompanied by data and phylogenetic analysis, respectively. Organisms were showing resistance against ampicillin (10 *μ*g), cefixime (5 *μ*g), ceftriaxone (30 *μ*g), nalidixic acid (30 *μ*g), ciprofloxacin (5 *μ*g), and ofloxacin (5 *μ*g) on antimicrobial susceptibility test. WGS were done on selected isolate which identified 25 virulence genes (*air*, *astA*, *chuA*, *fyuA*, *gad*, *hra*, *iha*, *irp2*, *iss*, *iucC*, *iutA*, *kpsE*, *kpsMII_K1*, *lpfA*, *mchF*, *ompT*, *papA_F43*, *sat*, *senB*, *sitA*, *terC*, *traT*, *usp*, *vat*, and *yfcV*) and seven housekeeping genes (*adk*, *fumC*, *gyrB*, *icd*, *mdh*, *purA*, and *recA*). Among resistance genes, seven genes (*TolC*, *emrR*, *evgA*, *qacEdelta1*, *H-NS*, *cpxA*, and *mdtM*) were identified to be involved in antibiotic efflux, three AMR genes (*aadA5*, *mphA*, and *CTX-M-15*) were involved in antibiotic inactivation, and two genes (*sul1* and *dfrA14*) were found to be involved in antibiotic drug replacement. Our data identified antibiotic resistance and virulence genes of the isolate. We suggest further research work to establish region-based resistance profile in comparison with the global resistance pattern.

## 1. Introduction

The World Health Organization (WHO) has issued a report highlighting common healthcare-associated bacterial organisms showing increased rates of antimicrobial resistance globally [1]. It was revealed in a study that the mortality rates can reach to around ten million per year by 2050 in the absence of any action taken against antimicrobial resistance [2].

Therefore, among the various resistance-causing organisms, *E. coli* has gained great attention globally after being a serious pathogen and with its diverse virulence capabilities [3].

Urinary tract infections (UTIs) are one of the most prevalent bacterial outpatient infections and frequently occur in women accounting for almost 50-60% infections in adult women and affecting 150 million people per annum globally [4, 5]. This high prevalence has been linked to several risk factors, including catheterization, surgical manipulation, and disruption of the urinary tract, mainly among diabetic and immunosuppressive patients, along with recurrent hospitalizations and other comorbidities [6]. UTIs are caused by various microorganisms but uropathogenic *Escherichia coli* (UPEC) being the primary cause (70%–90% of all UTIs) [7, 8]. In developing countries like Pakistan, it is a serious threat to the public health due to its association with high morbidity and mortality rate [9].

*E. coli* is a gram-negative organism and usually resides in the intestine of humans [10]. Structurally, the genome of *E. coli* strain varies in size between extragenetic elements, pathogenic variants, and commensal strains [11].

Animals and humans both have *Escherichia coli* in their intestines. Numerous strains include enteropathogenic *Escherichia coli* (EPEC), Shiga toxin-producing *Escherichia coli* (STEC), enterohaemorrhagic *Escherichia coli* (EHEC), enteroaggregative *Escherichia coli* (EAEC), enterotoxigenic *Escherichia coli* (ETEC), enteroinvasive *Escherichia coli* (EIEC), and diffusely adherent *Escherichia coli*(DAEC) [12].

Each strain possesses different virulence genes, including *eae*, *tir*, and *bfpA* by EPEC, and ap*AA* by EAEC. Two different toxins are reported in EHEC that include Stx1/Stx2 (Shiga-like toxin) and ehxA (enterohaemolysin), whereas, an invasion plasmid (vir regulon) is reported in EIEC. There are two enterotoxins heat-stable (S.T.) and labile-stable (L.T.) enterotoxins, by ETEC [12].

UPEC contains many genes that encode different virulence factors taking part to its pathogenesis including toxins, capsule, serum resistance, adhesive properties, and its iron uptake systems [13, 14]. Major virulence genes possessed by UPEC strains are aerobactin (*aer*), P fimbriae (*pap*), hemolysin (*hly*), type 1 fimbriae, afimbrial adhesin I (*afa I*), cytotoxic necrotizing factor 1 (*cnf 1*), S fimbriae (*sfa*), adhesins, and fimbriae. The other virulence genes are *kpsMT*, *ompT*, *usp*, *iroN*, *iha*, *set 1*, and *astA*, group II capsule synthesis; *sfa/foc*, *S*, and *F1Cfimbriae*; *iutA* and *traT*, serum resistance; and *fimH* [14, 15]. These virulence genes/factors possess crucial properties that allow microorganisms to establish its virulence properties effectively on its host to induce disease and also induce resistance, enabling them to cause pathogenesis impacting health, social, and economic issues even in the first world countries, like the UK, Norway, and Georgia [16–19].

Despite the knowledge of such a wide range of virulence genes of *E. coli*, antibacterial resistance is still a major concern around the globe [20]. The treatment for an organism has become progressively complicated because of the presence of resistance to usually the 1st-line antimicrobial drugs [21]. Antimicrobial resistance in UPEC has remarkably increased in the last few years and has become a tremendous public health issue [14, 22, 23]. UPEC strains were observed to be resistant mainly to trimethoprim-sulfamethoxazole, ampicillin, and ampicillin-sulbactam [21, 24]. *E. coli* can acquire AMR genes through mobile genetic elements (MGE), which include plasmids, integrons, gene cassettes, insertion sequences, and transposons [25]. A large number of resistance-encoding MGE, specific plasmids, are shared between different members of the *Enterobacteriaceae* and thus further promote the spread of resistance genes [26]. MGE can also indicate virulence factors and the interplay between virulence and antimicrobial resistance [23]. Therefore, it imposes the need to identify the genes which are responsible for the resistance to cater and improve the treatment outcome.

Most *E. coli* strains are isolated and identified by their O-antigen (lipopolysaccharide), H-antigen (flagellar), and K-antigen (capsular). The detailed structure of its genome and the responsible resistant genes can only be discovered through specialized molecular testing techniques, like whole genome sequencing [27, 28]. With the advent of sequencing technologies, it has become easier to get better insight into bacterial pathogenesis and to identify alternative therapeutics [29]. Therefore, the current study is aimed at characterizing a local MDR *E. coli* strain specifically associated with UTIs and at analyzing the genetic variants associated with it using the whole genome sequencing approach. In this study, we aim to identify the virulence and AMR profile of the local UPEC strain. Eventually, the sequenced genome will be used to get an insight into epidemiological studies of local *E. coli* strains which will help to get the global pattern using pangenomic approach as there is very less genome sequencing data available from Pakistan (especially from Balochistan). Moreover, the global burden of UTIs suggests detailed *in silico* analyses of UPEC, thus identifying possible therapeutic strategies.

The World Health Organization (WHO) has issued a report highlighting common healthcare-associated bacterial organisms showing increased rates of antimicrobial resistance globally [1]. It was revealed in a study that the mortality rates can reach to around ten million per year by 2050 in the absence of any action taken against antimicrobial resistance [2].

Therefore, among the various resistance-causing organisms, *E. coli* has gained great attention globally after being a serious pathogen and with its diverse virulence capabilities [3].

## 2. Materials and Methods

### 2.1. Bacterial Isolates and Antibiotic Susceptibility Testing

A total of 100 UPEC samples were collected from pyelonephritis patients at Bolan medical complex Hospital and Sandeman Provincial Hospital, Quetta. 44 clinical samples with positive MDR species of *E. coli* were transported to the Microbiology Laboratory, University of Balochistan. Samples were then cultured on the MacConkey agar and cystine-lactose-electrolyte-deficient agar (CLED). Gram-staining characteristics and standard biochemical tests, sulfide indole motility [30], triple sugar iron (TSI), urease, and citrate, were performed following the procedures described by Akbar et al. [31] and Ishaq et al. [32].

Antimicrobial susceptibility test (AST) was determined by disk diffusion method on the Mueller-Hinton agar plate as recommended by the Clinical and Laboratory Standards Institute (CLSI) 2019.

Amoxicillin and clavulanate (30 *μ*g), ampicillin (10 *μ*g), amikacin (30 *μ*g), aztreonam (30 *μ*g), gentamicin (10 *μ*g), ceftriaxone (30 *μ*g), nitrofurantoin (300 *μ*g), nalidixic acid (30 *μ*g), trimethoprim (25 *μ*g), cefixime (5 *μ*g), fosfomycin (50 *μ*g), ciprofloxacin (5 *μ*g), piperacillin/tazobactam (40 *μ*g), ertapenem (10 *μ*g), and ofloxacin (5 *μ*g) antibiotics were tested for AST. All those isolates which were found resistant to four or more than four antibiotics were considered MDR and included in the study for detailed analysis. Similarly, the *E. coli* strain resistant to more than five antibiotics was used in the study to be processed for whole genome sequencing. Tables 1–4 are shown.

### 2.2. DNA Extraction

Bacterial genomic DNA was extracted from the cultured isolate using CTAB method with few modifications [33]. The isolated bacterial colonies were mixed with CTAB buffer containing 0.2% *β*-mercaptoethanol and proteinase K (20 mg/mL) into the 1.5 mL safe lock tube. The mixture was incubated at 60°C for 30 minutes, and after that, chloroform-isoamyl alcohol (24 : 1) was added into the tube. The tube was centrifuged, and the aqueous phase was collected into the new tube. Isopropanol (equal volume) was added and incubated at 4°C for 30 minutes and centrifuged. DNA was collected as a pellet while the supernatant was discarded. The precipitated DNA was washed two times with 70% ethanol, and the DNA pellet was air-dried. The DNA pellet was dissolved in 1× TE buffer and stored at 4°C till further processing. The quality of genomic DNA was assessed using 1% agarose gel electrophoresis while the quantity was estimated by dsDNA high sensitivity kit by Qubit fluorometer [34].

### 2.3. DNA Sequencing and Assembly

DNA library of MDR *E. coli* EQ101 was prepared using the Nextera XT kit (Illumina, San Diego, CA, US) according to the manufacturer's guidelines. High molecular weight genomic DNA (5 ng) was fragmented (~300 bases) by transposomes. The adapters and indexes were ligated to both DNA ends, and then, amplification of the adapter-ligated library was performed by PCR. The amplified library was purified by Agencourt AMPure XP beads. The quantity of purified library was estimated by dsDNA HS Qubit kit while the size distribution of the library was evaluated by 3% agarose gel electrophoresis. The library was denatured and diluted to 16pM with a hybridization buffer (HT1) [35]. The diluted library was loaded into the sequencing cartridge for high-throughput sequencing using the MiSeq Illumina platform. The paired-end sequencing was carried out by 2 × 151 cycles using V3 flow cell.

### 2.4. In Silico Genome Characterization and Resistome Analysis

The paired-end sequencing data was obtained in the fastq format containing short sequencing reads. The sequenced reads were assembled using Unicycler v0.4.9, which uses information by SPAdes v3.13.0 for assembly followed by polishing the aligned reads using Pilon v1.8 with a minimum threshold of 300 bases of contig length. Contigs having a length of fewer than 500 bases were filtered out. The serotype of the sequenced isolate was confirmed by depositing the sequenced data in the Center for Genomic Epidemiology (http://www.genomicepidemiology.org) for *E. coli* using the web-based SerotypeFinder 2.0, serotyping tool by applying default parameters. FimH was identified using FimTyper 1.0, and virulence genes were identified using the VirulenceFinder 2.0 database with the following parameters: threshold for ID 90% and minimum length 60%. The assembled genome was annotated using RAST tool kit. The comprehensive antibiotic resistance database [36] package was used to predict the antimicrobial resistance genes [37]. Resistance gene identifier criteria were set to predict, strict, and complete genes only. VirulenceFinder 2.0 was used to find out the genes responsible for virulent mechanisms.

### 2.5. Pangenome Analysis

Here, 63 UPEC genomes were retrieved from the NCBI database (40 complete, 22 draft, and E101) and were annotated by Prokka using the default parameters [38]. The pangenome analysis and the core-genome SNP-based phylogenetic analysis were performed by PanRV [39] which uses Roary [40] for the pangenome estimation.

## 3. Results

All samples showed the presence of *E. coli* after being processed through culture and sensitivity. Organisms showing resistance against a list of antibiotics were selected as multi-drug-resistant.

The de novo whole genome assembly of *E. coli* EQ101 was done by Unicycler using SPAdes. The assembled genome comprised 918 contigs covering a total length of 5,764,348 bases with an average G+C content found around 50.89% and N50 was 9,699. The serotype of the sequenced isolate was identified as H18 with 100% identity of H type (fliC gene) with GenBank accession number AY250001, while no hit was identified for O-type genes. FimH64 was identified in the sequenced isolate, and the threshold was 95% using FimTyper 1.0.

Genome annotation of the assembled whole genome was carried out by PATRIC. The assembled genome consisted of 6,277 protein coding sequences (CDS), 53 transfer RNA (tRNA) genes, and 2 ribosomal RNA (rRNA) genes. The sequenced reads were aligned with the *E. coli* reference genome MG1655. The genome coverage of the sequenced isolate was found to be around 93.7%, and the mean depth coverage was 60.82×.

The annotation included 737 hypothetical proteins and 5,540 proteins with functional assignments. The proteins with functional assignments included 1,573 proteins with Enzyme Commission (EC) numbers, 1,304 with Gene Ontology (GO) assignments, and 1,104 proteins that were mapped to KEGG pathways. PATRIC annotation included two types of protein families, the genus-specific protein families (PLFams) which have 5,956 proteins sequenced genome and the cross-genus protein families (PGFams) involving 6,070 proteins.

A subsystem is a collection of proteins that combinedly implement a targeted biological process or structural complex and PATRIC. Numerous genes are involved in metabolisms followed by energy, protein processing, membrane transport, stress response, defense, virulence, cellular process, etc.

### 3.1. Pangenome-Based Phylogenetic Analysis

**Figure 1:**
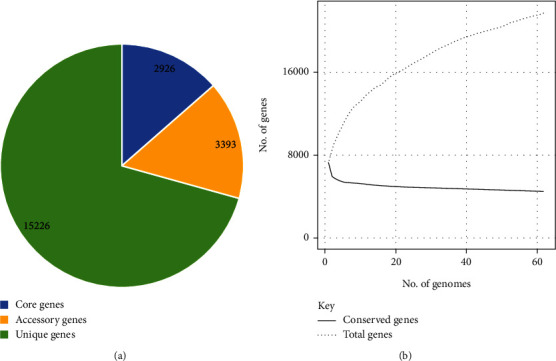
Pan-genome analysis of *E. coli* strains causing urinary tract infections in humans. (a) The pie chart shows a number of core, accessory, and unique genes in 63 genomes of UPEC strains. (b) The pangenome vs. core-genome plot of UPEC genomes.

The pangenome of selected UPEC strains consists of 21585 genes, of which 2,926 (13.55%) were core genes, 3,393 (15.7%) were accessory genes, and 15266 (70.7%) were unique genes (Figure 1(a)). The stats confirm the highly diverse nature of *E. coli* strains having an open pangenome.

**Figure 2:**
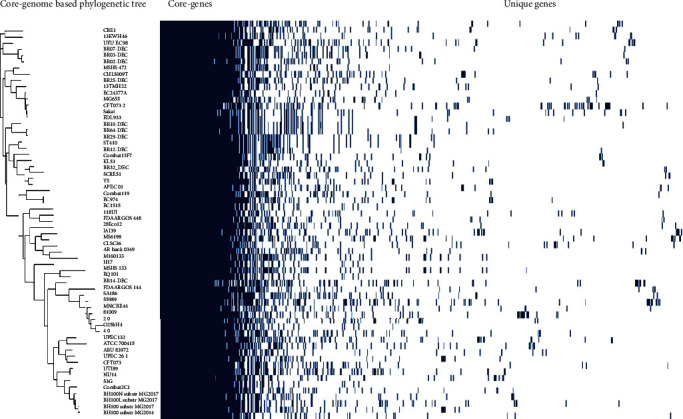
Core-genome SNP-based phylogenetic tree and heatmap of gene presence and absence in 63 UPEC strains.

**Figure 3:**
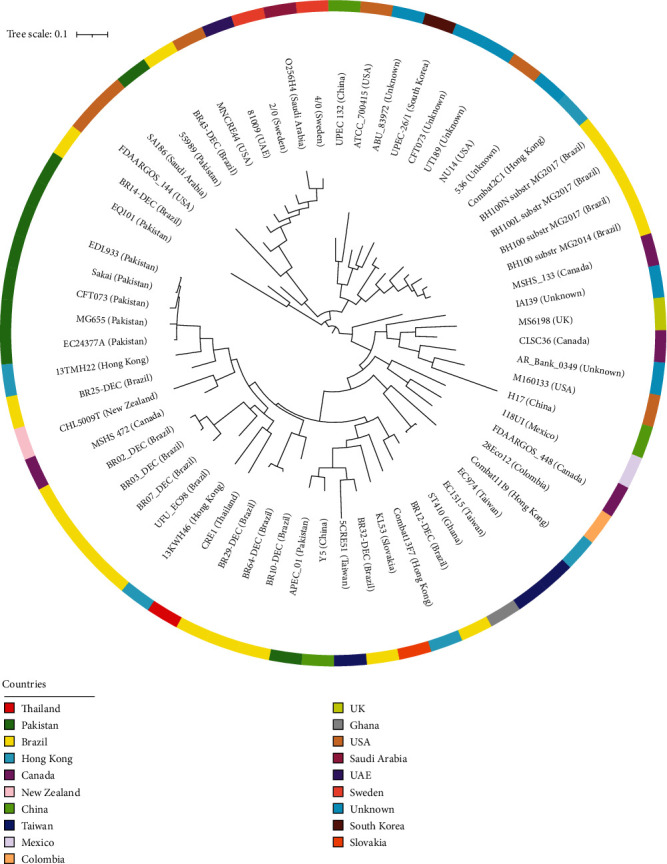
Circularized SNP tree of 63 global UPEC strains indicating same clade of EQ101 and BR-14 DEC. Most of the other Pakistani strains shared similar clade. Color key indicating countries is provided alongside the circular tree.

The core-genome-based phylogenetic analysis grouped the EQ101 with a Brazilian strain BR-14 DEC (Figures 2 and 3). Both the strains have 99.95% identity. The EQ101 genome did not contain any uniquely present or absent genes because of its contig level assembly.

### 3.2. Antibiotic Resistance Genes

**Figure 4:**
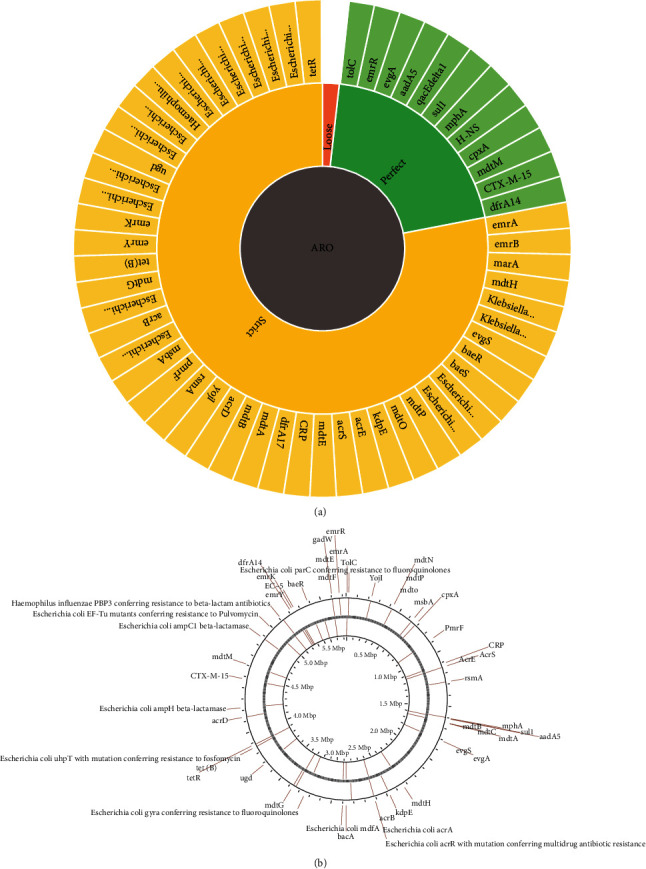
(a) Classification of antimicrobial resistance genes. (b) Antimicrobial resistance genes in EQ101.

We found a total of 58 AMR genes in the whole genome data of *E. coli* EQ101. Resistance gene identifier criteria were set to predict, strict, and complete genes only, which returned with 12 perfect hits and 46 strict hits with no lose hits. The resistance mechanism for the perfect RGI criteria includes seven genes *TolC*, *emrR*, *evgA*, *qacEdelta1*, *H-NS*, *cpxA*, and *mdtM*, involved in antibiotic efflux, and three AMR genes, i.e., *aadA5*, *mphA*, and *CTX-M-15*, involved in antibiotic inactivation, while two genes *sul1* and *dfrA14* involved in antibiotic drug replacement. The resistance mechanism in strict hit includes 35 genes involved in antibiotic efflux, two have reduced permeability to antibiotics, 3 involved in antibiotic inactivation, one involved in antibiotic target replacement, and 12 involved in antibiotic target alteration. The details of the AMR gene, drug class, and resistance mechanism are shown in Figures 4(a) and 4(b).

10 gene families and their respective AMR genes were found to have “perfect hits” (100% identity of matching region) including EC beta-lactamase (EC-5), trimethoprim-resistant dihydrofolate reductase dfr (dfrA14), CTX-M beta-lactamase (CTX-M-15), undecaprenyl pyrophosphate-related proteins (bacA), ANT(3^″^) (aadA5), sulfonamide-resistant sul (sul1), macrolide phosphotransferase [41] (mphA and MrX), resistance-nodulation-cell division (RND) antibiotic efflux pump (TolC, cpxA, evgA, h-NS, and gadW), major facilitator superfamily (MFS) antibiotic efflux pump (TolC, qacEdelta1, leuO, evgA, H-NS, mdtM, and emrR), and ATP-binding cassette (ABC) antibiotic efflux pump (TolC) with resistance to the following respective drug classes: diaminopyrimidine antibiotic, lincosamide antibiotic, nucleoside antibiotic, sulfonamide antibiotic, disinfecting agents and antiseptics, penem, phenicol antibiotic, rifamycin antibiotic, aminocoumarin antibiotic, peptide antibiotic, tetracycline antibiotic, penam, cephamycin, glycylcycline, cephalosporin, carbapenem, aminoglycoside antibiotic, fluoroquinolone antibiotic, and macrolide antibiotic.

14 gene families and their AMR genes were found to have “strict hits” (90-100% identity of matching region) which are as follows: penicillin-binding protein mutations conferring resistance to beta-lactam antibiotics (*Haemophilus influenzae* PBP3 conferring resistance to beta-lactam antibiotics), elfamycin-resistant EF-Tu (*Escherichia coli* EF-Tu mutants conferring resistance to pulvomycin), antibiotic-resistant UhpT (*Escherichia coli* UhpT with mutation conferring resistance to fosfomycin), fluoroquinolone-resistant parC (*Escherichia coli* parC conferring resistance to fluoroquinolones), general bacterial porin with reduced permeability to beta-lactams (marA and *Escherichia coli* soxS with mutation conferring antibiotic resistance), van ligase (vanG), glycopeptide resistance gene cluster (vanG), small multidrug resistance (SMR) antibiotic efflux pump (*Klebsiella pneumoniae* KpnE, *Klebsiella pneumoniae* KpnF, and *Escherichia coli* emrE), kdpDE (kdpE), trimethoprim-resistant dihydrofolate reductase dfr (dfrA17), resistance-nodulation-cell division (RND) antibiotic efflux pump (CRP, AcrE, AcrS, rsmA, mdtC, mdtB, mdtA, evgS, acrB, *Escherichia coli* acrA, acrD, marA, baeR, mdtE, mdtF, *Escherichia coli* acrR with mutation conferring multidrug antibiotic resistance, *Escherichia coli* soxR with mutation conferring antibiotic resistance, *Escherichia coli* soxS with mutation conferring antibiotic resistance, and *Escherichia coli* marR mutant conferring antibiotic resistance), pmr phosphoethanolamine transferase (PmrF and ugd), major facilitator superfamily (MFS) antibiotic efflux pump (mdtN, mdtO, mdtP, evgS, mdtH, *Escherichia coli* mdfA, mdtG, tet(B), emrY, emrK, emrA, *Escherichia coli* soxR with mutation conferring antibiotic resistance, *Escherichia coli* soxS with mutation conferring antibiotic resistance, and tetR), and ATP-binding cassette (ABC) antibiotic efflux pump (YojL, msbA, *Escherichia coli* soxR with mutation conferring antibiotic resistance, and *Escherichia coli* soxS with mutation conferring antibiotic resistance). The drug classes they show resistance against are elfamycin antibiotic, penem, carbapenem, monobactam, glycopeptide antibiotic, phosphonic acid antibiotic, aminoglycoside antibiotic, aminocoumarin antibiotic, diaminopyrimidine antibiotic, phenicol antibiotic, rifamycin antibiotic, tetracycline antibiotic, glycylcycline, cephamycin, cephalosporin, penam, fluoroquinolone antibiotic, macrolide antibiotic, nitroimidazole antibiotic, disinfecting agents and antiseptics, nucleoside antibiotic, and peptide antibiotic.

### 3.3. Virulence-Associated Genes

VirulenceFinder 2.0 identified 25 virulence genes (*air*, *astA*, *chuA*, *fyuA*, *gad*, *hra*, *iha*, *irp2*, *iss*, *iucC*, *iutA*, *kpsE*, *kpsMII_K1*, *lpfA*, *mchF*, *ompT*, *papA_F43*, *sat*, *senB*, *sitA*, *terC*, *traT*, *usp*, *vat*, and *yfcV*). *Air* (UniprotKB:P50466), *Iha* (UniprotKB:A0A061YB93), and *iutA* (UniprotKB:P14542) act as transmembrane receptors, and Gad system helps to maintain a near-neutral intracellular pH when cells are exposed to extremely acidic conditions, so the ability to survive transit through the acidic conditions of the stomach is essential for successful colonization of the mammalian host by commensal and pathogenic bacteria. *Sat* (UniprotKB:P13018) is involved in resistance to streptothricin, a broad-spectrum antibiotic, via acetylation of the beta amino group of the first beta-lysyl moiety of streptothricin. *traT* (UniprotKB:B1VCB1) is responsible for preventing unproductive conjugation between bacteria carrying like plasmids. *lpfA* (UniprotKB:Q8VPB9), *yfcV* (UniprotKB:P77288), and *sitA* (UniprotKB:Q6J5P0) play role in cell adhesion. *iutA* (UniprotKB:P14542), *mchF* (UniprotKB:Q9EXN5), *kpsE* (UniprotKB:P42501), *chuA* (UniprotKB:A0A0K3JCI0), *fyuA* (UniprotKB:Q798T6), and *irp2* (UniprotKB:A0A061JYT2) take part in cell transportation. *ompT* (UniprotKB:P09169) has aspartic-type and serine-type endopeptidase activity and take part in proteolysis.

MLSR analyzed seven *E. coli* housekeeping genes (*adk*, *fumC*, *gyrB*, *icd*, *mdh*, *purA*, and *recA*) through MLST 2.0 and identified *adk_14*, *fumC_14*, *gyrB_10*, *mdh_17*, *purA_7*, and *recA_10* alleles while no hit was found for *icd* allele. *Adk* (UniprotKB:P69441) plays an important role in cellular energy homeostasis and in adenine nucleotide metabolism. Icd (UniprotKB:P08200) and *fumC* (UniprotKB:P05042) are involved in the TCA cycle and response to oxidative stress. *gyrB* (UniprotKB:P0AES6) maintain chromosomes in an underwound state. *E. coli gyrase* has higher supercoiling activity than other characterized bacterial gyrases. *Mdh* (UniprotKB:P61889) catalyzes the reversible oxidation of malate to oxaloacetate, and *recA* (UniprotKB:P0A7G6) is required for homologous recombination, cellular response to DNA damage stimulus, and the bypass of mutagenic DNA lesions by the SOS response. The SOS response controls an apoptotic-like death (Oliveira et al.) induced in response to DNA damaging agents that is mediated by *RecA* and *LexA*. *Adk* (UniprotKB:P69441), *mdh* (UniprotKB:P61889), and *purA* (UniprotKB:P0A7D4) take part in energy metabolism.

*TolC* (UniprotKB:P02930) is an outer membrane channel, which is required for the function of several efflux systems such as AcrAB-TolC, AcrEF-TolC, EmrAB-TolC, and MacAB-TolC. These systems are involved in the export of antibiotics and other toxic compounds from the cell. *evgA* seems to control the expression of multidrug efflux operon. *H-NS* (UniprotKB:P0ACF8) is involved in bacterial chromosome organization, compaction and binds to the upstream and downstream regions of initiating RNA polymerase, trapping it in a loop and thereby, preventing the transcription process. It can also increase translational efficiency of mRNA with suboptimal Shine-Dalgarno sequences. Hydroxyisobutyrylation on Lys-121 decreases the DNA-binding activity of H-NS, promotes the expression of acid-resistant genes, and enhances bacterial survival under extreme acid stress.

*cpxA* (UniprotKB:P0AE82) responds to envelope stress response by activating the expression of downstream genes and is involved in several diverse cellular processes, including the functioning of acetohydroxyacid synthase I, the biosynthesis of isoleucine and valine, the TraJ protein activation activity for *tra* gene expression in F plasmid, and the synthesis, translocation, or stability of cell envelope proteins. *cpxA* is also involved in cell adhesion, so it takes part in biofilm formation. *mdtM* (UniprotKB:P39386) plays in cellular transportation and confers resistance to acriflavine, chloramphenicol, and norfloxacin. *emrR* (UniprotKB:C3SY57) and *evgA* (UniprotKB:P0ACZ4) play role in transcription. *CTX-M-15* (UniprotKB:W8YE54) has beta-lactamase activity and is involved in antibiotic resistance.

*sul1* (UniprotKB:P0C002) is implicated in resistance to sulfonamide. *dfrA14* (UniprotKB:B6SCG1) is a key enzyme in folate metabolism. The sul1 enzyme catalysed process is an essential reaction for de novo glycine and purine synthesis and DNA precursor synthesis.

## 4. Discussion

Genes related to the resistance to a certain type of antibiotics were the key aim of the present study. Therefore, the genes having roles in antimicrobial resistance were analyzed and compared with the phenotype of the resistance pattern.

The identified isolates of the study showed resistance mainly against amoxicillin, cefpirome, nalidixic acid, pipemidic acid, aztreonam, cefaclor, cefotaxime, ceftriaxone, ofloxacin, norfloxacin, ciprofloxacin, cephradine, cefuroxime, cefixime, ceftazidime, cefepime, and cotrimoxazole and showed genotype-phenotype correlations as well according to the aim of the study.

While discussing the various genes, the isolated traT virulence gene was observed to be involved in antimicrobial resistance. Its role was also supported by a previous study where Rezatofighi et al. [42] reported the significant prevalence of pathogenicity-associated island, *papAH*, *papEF*, *fimH*, *fyuA*, and *traT* genes in UPEC isolates. Another study revealed the presence of certain virulence genes including *iha*, *lpfA*, *aafC*, *nfaE*, *eilA*, *eae*, and *bfpA* for adherence to host cells. It further identified virulence factors including *senB*, *astA*, and *pic* that promote toxin production. Furthermore, toxins that promote *E. coli* protease production included *sat* and *vat* [43].

The variability of the housekeeping genes has been significantly identified and reported in a study that among the seven genes, *fumC* and *gyrB* presented the highest degree of nucleotide diversity and the greatest number of polymorphic nucleotide sites [3], suggesting to contribute to a high degree resistance pattern as identified in this study as well.

Among the identified resistance genes in the study, *mdtM* and *acEdelta1* were found to be associated with antibiotic efflux, which was further confirmed through a study and reported that the same genes were involved in causing multidrug resistance and helped the organism to tolerate higher concentrations of antibiotics, external pH, and stress conditions involving alkaline environment [44, 45]. Furthermore, *mphA* gene, which was observed to involve in antibiotic inactivation, was found to be responsible for causing resistance to azithromycin [46, 47]. Another *sul1* gene was observed to be responsible for antibiotic drug replacement in this study and was also found as a sulfonamide resistance gene in some other study [46, 47].

The rest of the identified genes were found to be involved in affecting antibiotic targets, and their overexpression resulted in reduced permeability and antibiotic efflux. The presence of such higher numbers of resistance gene cassettes in a single sequence indicates higher resistance patterns in the country and requires further sequence-based studies and population-based comparisons of such gene clusters across the globe to cope with antimicrobial resistance and increase the efficacy of the treatment outcome.

The pangenome analysis revealed that more than 70% of genes were part of a unique genome which confirms the high diversity in ubiquitous *E. coli* strains and thus more divergence in the phylogenetic relationship as also discussed in earlier studies [48–50].

## 5. Conclusion

The study concluded that the sequenced organism (*E. coli*) was chosen based on higher resistance to most of the available antibiotics and genotype-phenotype correlations. Among the number of genes, correlating with antibiotic resistance, certain virulence, housekeeping, and resistance genes were identified which were also present in other UPEC strains. Pangenome analysis confirmed *E. coli*'s ubiquitous nature and identified the closest phylogenetic relative of EQ101. Despite the identification of several genes involved in the resistance pattern, further research work would be required to establish a region-based resistance pattern in comparison with the global resistance pattern.

## Figures and Tables

**Table 1:** CLSI zone size.

CLSI zone sizes				
Antibiotic	Sensitive ≥	Intermediate	Resistant ≤	EQ101
Amoxicillin and clavulanate (30 *μ*g)	18	14-17	13	18
Ampicillin (10 *μ*g)	17	14-16	13	0
Amikacin (30 *μ*g)	17	15-16	14	21
Aztreonam (30 *μ*g)	21	18-20	17	23
Gentamicin (10 *μ*g)	15	13-14	12	15
Ceftriaxone (30 *μ*g)	23	20-22	19	0
Nitrofurantoin (300 *μ*g)	17	15-16	14	20
Nalidixic acid (30 *μ*g)	19	14-18	13	6
Trimethoprim (25 *μ*g)	16	11-15	10	17
Cefixime (5 *μ*g)	19	16-18	15	0
Fosfomycin (50 *μ*g)	16	13-15	12	22
Ciprofloxacin (5 *μ*g)	31	21-30	20	0
Piperacillin/tazobactam (40 *μ*g)	21	18-20	17	21
Ertapenem (10 *μ*g)	22	19-21	18	22
Ofloxacin (5 *μ*g)	16	13-15	12	8

**Table 2:** The phenotypic resistance profile of EQ101.

No.	Antibiotics	Sensitivity	Resistant	Intermediate
1	Ampicillin (10 *μ*g)	35%	60%	5%
2	Amoxicillin/clavulanic acid (30 *μ*g)	70%	26%	4%
3	Piperacillin/tazobactam (40 *μ*g)	80%	19%	1%
4	Cefixime (5 *μ*g)	60%	38%	2%
5	Ceftriaxone (30 *μ*g)	78%	21%	1%
6	Ertapenem (10 *μ*g)	55%	39%	6%
7	Amikacin (30 *μ*g)	51%	39%	10%
8	Gentamycin (10 *μ*g)	55%	39%	6%
9	Nalidixic acid (30 *μ*g)	35%	60%	5%
10	Ciprofloxacin (5 *μ*g)	45%	48%	7%
11	Ofloxacin (5 *μ*g)	34%	66%	0%
12	Fosfomycin (50 *μ*g)	60%	25%	15%
13	Nitrofurantoin (300 *μ*g)	80%	11%	9%
14	Sulfamethoxazole-trimethoprim (25 *μ*g)	37%	41%	22%
15	Aztreonam (30 *μ*g)	58%	30%	12%

**Table 3:** The genomic features and characteristics of the *E coli* strain EQ101.

Characteristics	EQ101
Genome size	5,764,348 bp
Contigs	918
G+C content	50.89%
N50	9,699
Standard deviation of contig lengths	8531.109786558065

**Table 4:** Phenotypic and genotypic resistance profile of EQ101.

No.	Antibiotics	PR	GR
1	Ampicillin (10 *μ*g)	R	*blaCTX-M-15*
2	Amoxicillin/clavulanic acid (30 *μ*g)	S	ND
3	Piperacillin/tazobactam (40 *μ*g)	S	ND
4	Cefixime (5 *μ*g)	R	*TolC*, *H-NS*, *CTX-M-15*, *EC-5*
5	Ceftriaxone (30 *μ*g)	R	*blaCTX-M-15*
6	Ertapenem (10 *μ*g)	S	*marA*, *Escherichia coli soxS* with mutation conferring antibiotic resistance
7	Amikacin (30 *μ*g)	S	*aadA5*
8	Gentamycin (10 *μ*g)	S	*aadA5*
9	Nalidixic acid (30 *μ*g)	R	*TolC*, *evgA*, *H-NS*, *mdtM*, *gadW*, *emrR*
10	Ciprofloxacin (5 *μ*g)	R	*TolC*, *evgA*, *H-NS*, *mdtM*, *gadW*, *emrR*
11	Ofloxacin (5 *μ*g)	R	*TolC*, *evgA*, *H-NS*, *mdtM*, *gadW*, *emrR*
12	Fosfomycin (50 *μ*g)	S	ND
13	Nitrofurantoin (300 *μ*g)	S	ND
14	Sulfamethoxazole-trimethoprim (25 *μ*g)	S	*sul1*, *dfrA17*, *dfrA14*
15	Aztreonam (30 *μ*g)	S	*marA*, *Escherichia coli soxS* with mutation conferring antibiotic resistance
16	Tetracycline	ND	*tet(B)*
17	MLS (macrolide, lincosamide, and streptogramin B)	ND	*mph(A)*
18	Disinfectant	ND	*qacE*

PR = phenotypic resistance; GR = genotypic resistance; R = resistant; S = susceptible; ND = not determined.

## Data Availability

All short reads and assemblies associated with this study are available at RAST, CARD, and PATRIC. The genomic data is present at NCBI https://www.ncbi.nlm.nih.gov/biosample/SAMN26578128/.

## Supplementary Materials

Supplementary Materials Supplementary Results, Table 1 and Table 2.
