# Supplementary material for: Virulence Factors and Antimicrobial Resistance of Uropathogenic Escherichia coli EQ101 UPEC Isolated from UTI Patient in Quetta, Balochistan, Pakistan
Source: Biomed Res Int. 2023 Sep 11;2023:7278070. doi: 10.1155/2023/7278070 (PMC10506881; doi:10.1155/2023/7278070)
Supplement: Supplementary Materials — Supplementary Results, Table 1 and Table 2. [file 7278070.f1.docx]

1. **You have to assess the quality of assemblies with tools like QUAST**

QUAST analysis was run at default parameters against *E. coli* strain K-12 sub-strain MG1655 reference genome and the results are as follows:

All statistics are based on contigs of size >= 500 bp, unless otherwise noted (e.g., "# contigs (>= 0 bp)" and "Total length (>= 0 bp)" include all contigs).

Aligned to "reference" | 4639675 bp | 1 fragment | 50.79 % G+C
4324 genomic features | 884 operons

**Supplementary Table 1 Quality assessment of EQ101 genome assemblies with QUAST**

| **Genome statistics** | **GCA_022604675.1_ASM2260467v1_…** |
| --- | --- |
| Genome fraction (%) | 87.253 |
| Duplication ratio | 1.016 |
| # genomic features | 3437 + 413 part |
| # operons | 588 + 214 part |
| Largest alignment | 49060 |
| Total aligned length | 4089439 |
| NG50 | 16308 |
| NG75 | 10701 |
| NA50 | 7537 |
| NGA50 | 10222 |
| NGA75 | 4319 |
| LG50 | 88 |
| LG75 | 177 |
| LA50 | 182 |
| LGA50 | 121 |
| LGA75 | 287 |
| **Misassemblies** |  |
| # misassemblies | 83 |
| # relocations | 83 |
| # translocations | 0 |
| # inversions | 0 |
| # misassembled contigs | 73 |
| Misassembled contigs length | 1248176 |
| # local misassemblies | 151 |
| # scaffold gap ext. mis. | 0 |
| # scaffold gap loc. mis. | 0 |
| # unaligned mis. contigs | 19 |
| **Unaligned** |  |
| # fully unaligned contigs | 225 |
| Fully unaligned length | 872851 |
| # partially unaligned contigs | 185 |
| Partially unaligned length | 748003 |
| **Mismatches** |  |
| # mismatches | 95239 |
| # indels | 989 |
| Indels length | 3859 |
| # mismatches per 100 kbp | 2352.59 |
| # indels per 100 kbp | 24.43 |
| # indels (<= 5 bp) | 862 |
| # indels (> 5 bp) | 127 |
| # N's | 0 |
| # N's per 100 kbp | 0 |
| **Statistics without reference** |  |
| # contigs | 736 |
| # contigs (>= 0 bp) | 738 |
| # contigs (>= 1000 bp) | 733 |
| # contigs (>= 5000 bp) | 351 |
| # contigs (>= 10000 bp) | 195 |
| # contigs (>= 25000 bp) | 36 |
| # contigs (>= 50000 bp) | 2 |
| Largest contig | 69339 |
| Total length | 5732989 |
| Total length (>= 0 bp) | 5733813 |
| Total length (>= 1000 bp) | 5731215 |
| Total length (>= 5000 bp) | 4784911 |
| Total length (>= 10000 bp) | 3674810 |
| Total length (>= 25000 bp) | 1280143 |
| Total length (>= 50000 bp) | 120824 |
| N50 | 13672 |
| N75 | 7037 |
| L50 | 125 |
| L75 | 269 |
| GC (%) | 50.84 |
| **Similarity statistics** |  |
| # similar correct contigs | 0 |
| # similar misassembled blocks | 0 |

1. **Was it BLAST search? What coverage, identity threshold was used?**

Yes, it was a Blast search. We used Resistance Gene Identifier of CARD to predict resistome from genome based on homology and SNP models. It was run on default parameters with the selection criteria perfect and strict hits only, nudges excluded and high-quality sequence/coverage. The results obtained with default parameters were:

**Supplementary Table 2 Coverage, identity and threshold used for BLAST search**

| No. | Hit | Criteria | Bit-score | Cut-Off | Percent Identity |
| --- | --- | --- | --- | --- | --- |
| 1 | TolC | Perfect | 924.9 | 900 | 100 |
| 2 | mphA | Perfect | 594.7 | 500 | 100 |
| 3 | Mrx | Perfect | 740 | 600 | 100 |
| 4 | evgA | Perfect | 400.6 | 390 | 100 |
| 5 | H-NS | Perfect | 258.1 | 240 | 100 |
| 6 | gadW | Perfect | 481.5 | 470 | 100 |
| 7 | mdtM | Perfect | 781.2 | 700 | 100 |
| 8 | gadW | Perfect | 481.5 | 470 | 100 |
| 9 | emrR | Perfect | 349 | 280 | 100 |
| 10 | cpxA | Perfect | 891.3 | 890 | 100 |
| 11 | aadA5 | Perfect | 527.7 | 450 | 100 |
| 12 | sul1 | Perfect | 549.7 | 500 | 100 |
| 13 | qacEdelta1 | Perfect | 224.9 | 190 | 100 |
| 14 | leuO | Perfect | 629 | 500 | 100 |
| 15 | bacA | Perfect | 525.4 | 500 | 100 |
| 16 | CTX-M-15 | Perfect | 557.4 | 500 | 100 |
| 17 | dfrA14 | Perfect | 320.9 | 300 | 100 |
| 18 | EC-5 | Perfect | 780.8 | 725 | 100 |
| 19 | emrR | Perfect | 349 | 280 | 100 |
| 20 | YojI | Strict | 1059.3 | 1050 | 99.63 |
| 21 | mdtN | Strict | 632.9 | 600 | 98.54 |
| 22 | mdtO | Strict | 1319.7 | 1300 | 98.1 |
| 23 | mdtP | Strict | 908.7 | 875 | 97.95 |
| 24 | msbA | Strict | 1094.7 | 1000 | 99.66 |
| 25 | PmrF | Strict | 627.9 | 550 | 99.38 |
| 26 | CRP | Strict | 419.5 | 400 | 99.52 |
| 27 | AcrE | Strict | 729.9 | 675 | 99.22 |
| 28 | AcrS | Strict | 435.3 | 380 | 98.64 |
| 29 | rsmA | Strict | 102.4 | 100 | 95.25 |
| 30 | dfrA17 | Strict | 316.2 | 300 | 99.36 |
| 31 | mdtC | Strict | 1899.8 | 1800 | 98.63 |
| 32 | mdtB | Strict | 1907.1 | 1800 | 99.81 |
| 33 | mdtA | Strict | 773.5 | 725 | 99.04 |
| 34 | evgS | Strict | 2365.1 | 2300 | 99.16 |
| 35 | mdtH | Strict | 781.9 | 750 | 99.75 |
| 36 | kdpE | Strict | 448.7 | 400 | 99.56 |
| 37 | acrB | Strict | 1988 | 1900 | 99.9 |
| 38 | Escherichia coli acrA | Strict | 747.7 | 670 | 99.5 |
| 39 | Klebsiella pneumoniae KpnE | Strict | 176.4 | 150 | 92.2 |
| 40 | Klebsiella pneumoniae KpnF | Strict | 189.5 | 150 | 94.4 |
| 41 | Escherichia coli mdfA | Strict | 760.4 | 700 | 96.83 |
| 42 | mdtG | Strict | 731.5 | 700 | 100 |
| 43 | ugd | Strict | 757.3 | 700 | 99.48 |
| 44 | tet(B) | Strict | 760.8 | 700 | 99.25 |
| 45 | acrD | Strict | 1976.8 | 1900 | 99.81 |
| 46 | vanG | Strict | 264.2 | 250 | 98.23 |
| 47 | marA | Strict | 260 | 230 | 99.21 |
| 48 | emrY | Strict | 986.5 | 900 | 99.61 |
| 49 | emrK | Strict | 663.3 | 600 | 98.29 |
| 50 | baeR | Strict | 481.9 | 450 | 99.58 |
| 51 | Escherichia coli emrE | Strict | 221.1 | 190 | 98.18 |
| 52 | mdtE | Strict | 721.1 | 675 | 99.48 |
| 53 | mdtF | Strict | 1934.5 | 1850 | 99.32 |
| 54 | emrA | Strict | 739.6 | 675 | 99.74 |
| 55 | Escherichia coli parC conferring resistance to fluoroquinolones | Strict | 1469.1 | 1400 | 99.87 |
| 56 | Escherichia coli UhpT with mutation conferring resistance to fosfomycin | Strict | 893.6 | 850 | 99.78 |
| 57 | Escherichia coli EF-Tu mutants conferring resistance to Pulvomycin | Strict | 785.8 | 700 | 99.75 |
| 58 | [Haemophilus influenzae PBP3 conferring resistance to beta-lactam antibiotics](https://card.mcmaster.ca/rgi/results/uuG7cZeEUFSbGTkNbbcxaRRpzP2icXbaXzz6h5X3) | Strict | 574.3 | 500 | 93.29 |
| 59 | Escherichia coli acrR with mutation conferring multidrug antibiotic resistance | Strict | 426.8 | 375 | 100 |
| 60 | Escherichia coli soxR with mutation conferring antibiotic resistance | Strict | 307.8 | 300 | 99.35 |
| 61 | Escherichia coli soxS with mutation conferring antibiotic resistance | Strict | 219.2 | 200 | 100 |
| 62 | tetR | Strict | 422.2 | 400 | 99.52 |
| 63 | Escherichia coli marR mutant conferring antibiotic resistance | Strict | 285.4 | 210 | 97.92 |

1. **Explain what is a lose hit and a strict hit in the method and if you were looking at the origin or resistance to be acquired or chromosomal, it should be mentioned in the results. You should use ResFinder to look for acquired resistance genes and then compared with CARD results**

The ‘Loose’ algorithm of CARD works outside of the detection model cut-offs to provide distant homologs of AMR genes and it also provides hits that may not have a role in AMR. In contrast, the ‘Strict’ algorithm detects previously unknown variants of known AMR genes, using detection models with curated similarity cut-offs to ensure the detected variant is likely a functional AMR gene.

To identify acquired antimicrobial resistance genes and chromosomal point mutations, the assembled genome of EQ101 was run against *Escherichia coli* at ResFinder 4.1 available at <https://cge.food.dtu.dk/services/ResFinder/> with default parameters (90% threshold for ID and 60% for minimum length). The results obtained were comparable to that of CARD results showing resistance against beta-lactams, quinolones, folate pathway antagonist, quartenry ammonium compounds, macrolides and tetracyclines due to the presence of *blaCTX-M-15*, *gyrA*, *sul1*, *dfrA17*, *qacE,* *mphA* and *tet(B)* genes. Point mutations were detected *in parE, parC, gyrA, pmrB, pmrA, 16S_rrsH, ampC, gyrB, 16S_rrsB, 16S_rrsC* and *23S* genes.
